# Southeast Asia's blind spot in reproductive health: from legal measures to lived access

**DOI:** 10.1016/j.lansea.2026.100856

**Published:** 2026-09-11

**Authors:** The Lancet Regional Health – Southeast Asia

Over the past decade, there has been remarkable progress in women's health in the WHO southeast Asia region. However, a recent Global Burden of Disease analysis highlights that progress has been stalled in maternal mortality, and several countries are off-track to achieve the Sustainable Development Goal target of fewer than 70 maternal deaths per 100,000 livebirths. This signals an urgent need for course correction.

Provision of expanded and improved sexual and reproductive health (SRH) services could reduce maternal deaths by about two-thirds, newborn deaths by 66%, and new HIV infections among babies aged 6 weeks and younger by 88%. Across the southeast Asia region, progress in SRH continues to be defined in terms of improved contraceptive usage and reduced fertility. A report by the Guttmacher Institute in 2019 highlighted key gaps in south Asia, with an unmet need for contraception being higher for adolescents aged 15–19 years than for all women aged 15–49 years (60% versus 29%). The report also marks that 16 million women had abortions in unsafe conditions and 23 million did not receive the treatment they need for sexually transmitted infections, in 2019.

These gaps reflect a persistent blind spot in understanding women's reproductive health needs in the region. National surveys generate estimates for the unmet need for contraception from data collected on contraceptive use among married women, excluding unmarried and adolescent populations. Surveys also fail to capture other components like abortion, unwanted pregnancies, sexually transmitted infections, infertility, and reproductive tract diseases such as polyendocrine metabolic ovarian syndrome and endometriosis. Additionally, health system constraints and prevailing cultural sensitivities in the region are a barrier in collecting such data. This is compounded by social factors such as intimate partner violence, which shapes both women's ability to access SRH services and their willingness to disclose related needs.

Primary research on reproductive health has the potential to provide the needed evidence for action and policy change. In this issue of *The Lancet Regional Health – Southeast Asia*, a systematic review and meta-analysis by Naqvi and colleagues highlights that women's education, employment, access to services, counselling by health workers, and spousal involvement influence contraceptive usage among reproductive age females in south Asia. Another set of five publications from Nepal Turn away study includes a longitudinal cohort of women seeking abortion at 22 facilities in Nepal who were followed up for 5 years. The research aims to provide evidence on how abortion denial affects physical health, antenatal care, birth outcomes, intimate partner violence, further reproductive decisions and women's empowerment.

While countries such as India, Nepal, and Bangladesh have relatively liberal abortion laws, their inconsistent implementation often leaves young women underserved. The findings from the Nepal Turn away cohort underscore key gaps in policy translation, with 15% of the women reporting unwanted birth and 33% reporting denial of abortion at first contact; one-fifth of these women reported experiencing partner violence. In India, 78% of abortions among adolescents are unsafe, and service delivery is undermined by provider bias, health system gaps, and social stigma. Bhutan, Maldives, Sri Lanka, and Myanmar, by contrast, retain restrictive laws, which are further influenced by other health system constraints.

Transitioning demographics, increase in premarital sexual activity, and evolving behavioural norms are driving greater demand for contraception and abortion services across the region. Most existing national programmes operate vertically, catering to a particular population group or health component. For example, family planning services are largely offered to married women with minimal integration across services—leaving a wide gap in access for unmarried and adolescent population. For most women, the first point of contact for a sexual and reproductive health service would be around childbirth.

Women navigate a lifelong continuum of physiological hormonal changes; SRH services should be designed bearing this in mind, addressing women's needs from menarche through post-menopause, by building synergies with existing health infrastructure rather than creating parallel systems. Sustainable gains in SRH require systems that prioritise women's agency and choice, empowering them as active decision makers rather than passive recipients of care. The gap between legal protection and actual service provision is largest for adolescents and unmarried young women. Countries that have led on progressive reproductive laws must now lead on implementation—because young women need services they can actually reach, not laws alone.

